# Supplementary material for: Ursodeoxycholic Acid Binds PERK and Ameliorates Neurite Atrophy in a Cellular Model of GM2 Gangliosidosis
Source: Int J Mol Sci. 2023 Apr 13;24(8):7209. doi: 10.3390/ijms24087209 (PMC10138647; doi:10.3390/ijms24087209)
Supplement: Supplementary file 1 [file ijms-24-07209-s001.zip › ijms-2259526-supplementary.pdf]

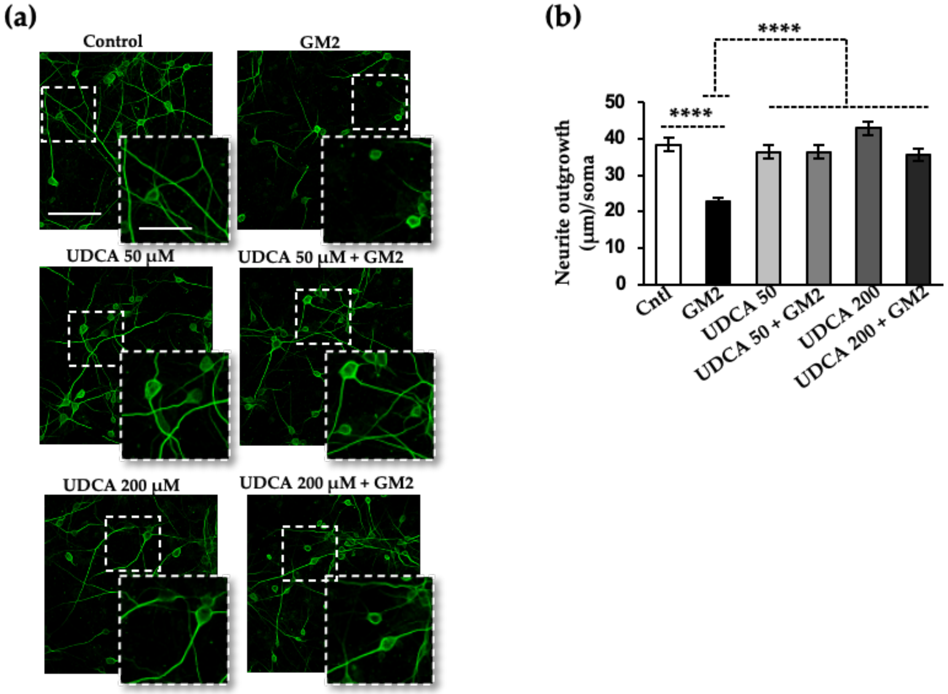


**Figure S1:** *Pre-incubation of cells with UDCA reduces neuritic atrophy induced by GM2 accumulation*. (**a**) Primary cortical neurons (10 DIV) were treated either with the vehicle, 50 μM or 200 μM of UDCA for 1 h, and then with or without 2 μM of GM2 for 19 h. The cells were fixed with 4% paraformaldehyde, immunolabeled with anti-MAP2 antibody, and visualized with Alexa Fluor-488 conjugated secondary antibody. Images were recorded with a confocal microscope. Scale bars: 100 μm (regular images), 50 μm (magnified images). (**b**) Histogram (mean ± SEM) represents neurite outgrowth with respect to total cells, analyzed with Image J plug-ins (NIH, USA).

****$p \leq 0.00001$ with respect to the control as determined by one-way ANOVA, n=3.

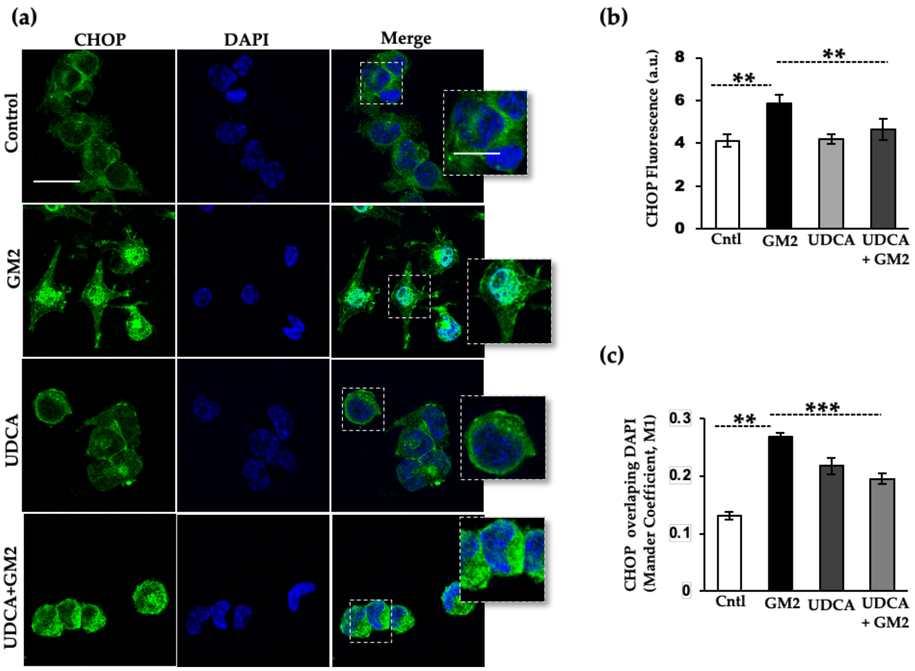


**Figure S2:** *UDCA reduces CHOP levels and its translocation to the nuclei in GM2-stressed cells.* (**a**) N2a cells were treated with 1000 µM of UDCA and incubated with or without exogenous GM2 (2 µM). Then, they were fixed, immunolabeled with anti-CHOP antibody, and visualized with Alexa Fluor-488 conjugated secondary antibody (green). Nuclei were labeled with DAPI (blue). Scale bars: 30 µm (regular images),15 µm (magnified images). Images were obtained with a confocal microscope (Zeiss LSM 800). (**b)** Histograms (mean ± SEM) represent green fluorescence intensity values, n=3. (**c**) Manders' overlap coefficient, M1 was calculated (Channel 1: green, Channel 2: blue). Histograms (mean ± SEM) represent M1 values, n=3. (**b**) and (**c**) ** $p \leq 0.001$ and ***, $p \leq 0.0001$ as determined by one-way ANOVA, n=3
